# Artificial intelligence as treatment support in breast cancer: current perspectives

**DOI:** 10.1016/j.breast.2025.104564

**Published:** 2025-08-22

**Authors:** Stefan Lukac, Florian Putz, Giacomo De Micheli, Chiara Corti, Wolfgang Janni, Sara M. Tolaney, Giuseppe Curigliano, Sibylle Loibl, Paolo Tarantino, Jose Pablo Leone

**Affiliations:** a Department of Obstetrics and Gynecology, University Hospital Ulm, Ulm, Germany; b Department of Radiation Oncology, Friedrich-Alexander-University Erlangen-Nürnberg, Erlangen, Germany; c Vita-Salute San Raffaele University, Milan, Italy; d Dana-Farber Cancer Institute, Harvard Medical School, Boston, MA, USA; e European Institute of Oncology IRCCS, Milan, Italy; f University of Milano, Milan, Italy; g German Breast Group, Neu-Isenburg, Germany

**Keywords:** Artificial intelligence, Breast cancer treatment, Surgery, Irradiation, Systemic treatment, Supportive care

## Abstract

With the increasing amount of information related to breast cancer (BC) management, artificial intelligence (AI) has emerged as a tool with the potential to enhance the quality of treatment through the efficient integration of large datasets; however, the specific areas for which AI may be ready for clinical implementation remain unclear. In this narrative review, we recapitulate the available data on AI utilization in BC treatment by focusing on surgical therapy, radiation therapy, systemic and supportive treatment, but including the diagnostics, too. While AI has been implemented successfully in mammography screening, preoperative consultation, and radiation oncology, its use intraoperatively, post-operatively, and in systemic and supportive treatment is still in development. AI has potential to improve care, but since the accuracy of AI varies, careful consideration of its benefits and limitations is necessary.

## Highlights

• AI enhances radiotherapy planning, reducing expert time and improving consistency.
• Surgical AI supports planning and margin assessment, needing broader validation.
• AI can aid systemic therapy decisions, though guideline alignment varies.
• Supportive care chatbots enhance self-care, but accuracy must be ensured.
• Bias mitigation and validation are crucial for AI integration in breast cancer care.

## 1 Introduction

The available knowledge about breast cancer (BC) treatment nowadays is enormous and growing rapidly by approximately 3000 new publications related to BC appearing every month [1]. This large and rapidly expanding amount of information challenges human capabilities to remain updated and be able to integrate all the relevant data points for optimal clinical decision-making. Artificial intelligence (AI) is already in use in various areas of medicine, with no exception to BC management. In this setting, recent technical advancements including AI promise to aid in data integration and interpretation. Therefore, the goal of our work is to provide the current state of the art of clinical application of AI as support in daily routine with a focus on the treatment of patients with BC.

## 2 Search strategy and selection criteria

Relevant literature for this review was identified through a targeted search in PubMed using the following keywords in publications up to April 2024: “breast cancer”, “breast carcinoma,” “malignant neoplasm of the breast,” in combination with terms like “surgery,” “margin evaluation,” “lumpectomy,” “mastectomy,” “radiation therapy,” “radiotherapy,” “brachytherapy,” “irradiation,” “systemic therapy,” “chemotherapy,” “targeted therapy,” “endocrine therapy,” “immunotherapy,” and “supportive treatment,” “supportive care,” “palliative care,” along with “artificial intelligence,” “AI,” “machine learning,” “deep learning,” or “neural network.” Only articles published in English were considered. The selection of references was guided by their originality and relevance to the thematic focus of each section of this review, ensuring a comprehensive and critical synthesis of the most pertinent contributions in the field. A narrative review was conducted to provide a focused, in-depth discussion of selected manuscripts considered particularly relevant to the current landscape of AI. This approach was selected to enable a broader perspective and interpretive synthesis of the state of the art, rather than an exhaustive cataloging of the literature. Although systematic reviews are appropriate for questions requiring comprehensive identification of all published evidence, they are less suited to perspective-driven analyses. The limitations of the narrative review approach, including the potential omission of relevant studies, are acknowledged.

## 3 Artificial intelligence and breast surgery

### 3.1 Preoperative period

Proper diagnostics are crucial for the preoperative consultation, especially considering oncological, surgical and cosmetic aspects. Some studies focused on the prediction of pathologic complete response (pCR) with the support of AI in patients receiving neoadjuvant chemotherapy (NAC) in order to omit surgery. The results reveal that even the best AI studied, a neural network (NN)-based model, showed low specificity of only 65.2 % (15/23), a false negative rate of 0 % (0/27), and the sensitivity was not reported [[2], [3], [4]]. These results need further evaluation but open a new field for AI in the planning of breast surgery [5].

The omission of axillary surgery in selected populations to de-escalate loco-regional treatment is currently under discussion. Based on 10 selected clinico-pathological parameters without image evaluation, the web-based artificial NN decision support tool (NILS) achieved a sensitivity of 90 % and specificity of 34 % for prediction of axillary lymph node involvement [6]. When comparing models for axillary ultrasound using standard statistical logistic regression and AI, the best prediction was provided by logistic regression, with a sensitivity of 95 % and a specificity of 46 %. However, AI achieved the highest accuracy at 92 %, with a sensitivity of 94 % and a specificity of 73 % [7]. Finally, if the DL-based model predicted axillary involvement based on images from positron emission tomography/computed tomography in early BC, it was not able to outperform the clinicians. However, the combination of AI and human evaluation increased the sensitivity by 10 % with unchanged specificity of 99 % [8].

In the planning of breast reconstruction, Chat Generative Pre-training Transformer (ChatGPT) 3.5 was compared with the three guidelines of the American Society of Plastic Surgeons regarding advice for post-mastectomy reconstructive surgery. ChatGPT failed to provide sufficiently accurate recommendations in comparison to these guidelines [9]. On the other hand, ChatGPT was able to provide sufficiently accurate information to the layperson, which was mostly a superficial overview [10].

Conversely, AI was able to identify more key perforators in angio-computed tomography for deep inferior epigastric perforator flap than the radiological team (183 vs 180) and could consequently save 2–3 h of time for a radiologist [11]. AI also possesses the ability to detect key features of the breast as it has achieved 97.7 % success in the identification of breast boundaries, nipple-areola complex and suprasternal notch in the study by Kenig et al. [12] Additionally, BreastGAN, a portable AI tool, was able to generate realistic breast images that were almost indistinguishable from the postoperative results of 309 patients after breast augmentation [13]. The CINDERELLA Trial will focus on evaluating preoperative consultations for patients with BC, including the AI-based generation of images to show possible postoperative outcomes [14].

### 3.2 Intraoperative period

The goal of BC surgery is a complete resection of the tumor. Intraoperative margin status prediction supported by AI could be based on specimen mammography evaluation as in the study by Chen et al. [15] The best AI presented sensitivity of 84 % and specificity of 42 %, which was similar to the data achieved in the interpretation by radiologist or surgeon. Another option is the application of AI-based sonography which achieved sensitivity of 95 % and specificity of only 67 % in a cohort of 52 patients with early BC in the study by Hu et al. [16] and sensitivity of 96 % and a specificity of 76 % in the study by Velupponar et al. [17] in predicting a close margin when compared to histology results. The new technique of ultraviolet fluorescence scanning microscopy of the whole surface of the resected specimen combined with DL could accurately localize areas that contain malignant or normal/benign tissue with a sensitivity of 100 % and specificity of 87.5 %. However, this method requires technical equipment and expertise which limits its implication in everyday surgeries [18].

### 3.3 Postoperative period

To identify risk factors for postoperative complications in breast reconstruction with an abdominal flap, Myung et al. [19] applied AI-based models. NNs provided the most effective prediction of risk factors, achieving an accuracy of 81 %, a sensitivity of 79 %, and a specificity of 89 %. Considering the complication rate with implant-based reconstructive surgery, a machine-learning model (MLM) predicted periprosthetic infection with an accuracy up to 82 % and risk of explantation of 84 % [20].

AI was previously tested in the prediction of postoperative pain and need for opioids in surgeries performed in an ambulatory setting, including surgeries for BC [21,22], with an accuracy of around 70 % when adjacent opioid requirement categories were aggregated [21]. Moreover, AI could identify risk factors for the postoperative neuropathic pain evaluated on the Douleur Neuropathique - 4 screening questionnaire [22].

## 4 Artificial intelligence and radiotherapy

### 4.1 Decision support in radiation oncology

Using a training dataset of patients who underwent mastectomies from the Surveillance, Epidemiology, and End Results (SEER) Program database, Jin et al. [23] found that a NN model outperformed conventional Cox regression for prognosis prediction. By predicting survival with and without post-mastectomy radiotherapy for novel patients, the authors were able to create a treatment recommendation system for identifying patients with hormone receptor-positive (HR+)/human epidermal growth factor receptor 2-negative (HER2-) T1/2N1M0 BCs who benefit from adjuvant radiation [23].

Large language models (LLMs) have also received interest in radiotherapy decision support. In a recent evaluation, Huang et al. [24] assessed the performance of GPT-4 for an American College of Radiotherapy exam as well as for difficult clinical cases from the Red Journal Grey Zone series. GPT-4 achieved 63 % correct answers for the BC radiotherapy questions in the exam and an average rank of 4.3 for the clinical radiotherapy cases compared to 3.3 for clinical experts [24]. Further improvement could be expected from dedicated medical LLMs as well as by including external information like guideline texts via in-context learning [25,26].

### 4.2 Radiotherapy treatment planning

The first step in BC radiotherapy treatment planning involves contouring of the organ at risk (OARC), like lungs, heart, contralateral breast and esophagus, which need to be spared from the radiotherapy dose. A DL model integrated into a commercial product for BC radiotherapy treatment planning required major correction for only 2 % of predicted OARC and enabled fully automatic OARC in 1.5 min [27]. The integrated AI had even shorter autocontouring times of fewer than 10 s, depending on other factors [[28], [29], [30]]. Clinically, DL auto-OARC can be directly used for treatment planning without any manual editing in the majority of cases and OAR structures [27,31,32]. Moreover, DL models have also been successfully developed and applied for delineation of difficult and low-contrast anatomical structures, like cardiac substructures for improved sparing of the heart [28,33,34]. However, DL-based auto-OARC always requires manual review, as corrections are necessary for a subset of cases. As a support tool, AI-supported autocontouring can significantly reduce the required expert time for BC radiotherapy OARC [31,35,36]. In a recent multicenter evaluation across 31 institutions, the availability of DL-based autocontouring significantly reduced inter-expert variations for OARC in BC radiotherapy [36].

Delineation of the target volume (TV) is the next crucial step during BC radiotherapy treatment planning. DL-based autosegmentation models like U-Net variants can be similarly employed for TV autocontouring to OARC auto-delineation. Moreover, some models are even trained to predict OARC and TV at the same time [29]. Aside from reducing the required expert time for treatment planning by around 25 min [31,37], DL-based TV auto-delineation is particularly interesting for improving standardization in BC radiotherapy [36]. In the large 31-center evaluation by Choi et al., [36] AI assistance improved inter-expert agreement for TV up to 19 %, even more than for OARC. Similarly, Buelens et al. [37] found an improvement in guideline consistency from 77.1 % to 90.7 % for DL autosegmentation compared to manual TV contouring. DL autocontouring can be applied to whole-breast radiotherapy treatment planning after lumpectomy [27] and mastectomy [38] as well as for stereotactic partial breast irradiation [39]. In addition, DL-powered targeting and auto-OARC has been applied as part of an automated radiotherapy treatment planning solution (Radiation Planning Assistant) to enable access and increase quality of BC radiotherapy in low-resource countries [40].

After the detection of OARC and defining the TV, the physical treatment planning is needed to find the optimal radiotherapy plan for the patient. AI can be also supportive in this area [41] as the goal of automatic treatment planning in BC is to reduce the manual time required, while ensuring high plan quality and improving standardization [[42], [43], [44], [45], [46], [47], [48]]. Kneepkens et al. [44] assessed AI-generated breast tangential-beam intensity modulated radiotherapy (IMRT) treatment plans against physician-generated treatment plans. Of the 20 plans, 95 % of DL-generated plans were rated acceptable in a blinded evaluation by four expert radiation oncologists while only 90 % of physician-generated plans were rated acceptable. Moreover, in another study for left-sided breast radiotherapy, DL-automated treatment planning was dosimetrically superior to conventional knowledge-based planning [49].

### 4.3 RT delivery

AI models have been successfully developed to support clinical experts in selecting the best technique for BC radiotherapy. Koide et al. [50] described a 2D convolutional NN (2D-CNN) to predict patients who benefit from deep inspiration breath hold (DIBH) radiotherapy from chest X-ray images alone. The authors found a sensitivity of 86 %, specificity of 91 % and an AUC of 0.86 for identifying patients with a mean heart dose reduction of more than 1 Grey for DIBH vs. conventional postoperative breast radiotherapy [50]. In a different approach, Kamizaki et al. [51] investigated three conventional MLMs as well as a deep NN for identifying patients with BC who do not benefit from DIBH. Using six clinical input parameters including chest wall thickness and tumor site as input parameters, the deep NN achieved an AUC of 0.88 and was superior to all conventional MLMs [51].

For surface-guided radiotherapy (SGRT), a region of interest (ROI) needs to be defined for proper patient positioning and monitoring. This manual process can be automated by DL models in BC radiotherapy [52,53]. Cui et al. [53] were able to show that DL-based automation for SGRT in BC can speed up the manual and operator-dependent ROI-definition process from 120 s to 1.2 s, while improving overall ROI quality.

DL applications can also be used to improve quality of cone beam computed tomography (CBCT) acquired at linear accelerators (LINAC) for BC radiotherapy, which suffer from image noise, X-ray scattering, artifacts and reduced field-of-view [[54], [55], [56], [57]]. Additionally, these AI-synthesized CT images can also be directly used for accurate dose calculation in BC treatment [54,56] and missing anatomical information from CBCTs can be restored with conditional GAN DL models using knowledge from the previous planning CT [57]. These AI extended images can then be used for accurate dose calculation and could improve patient positioning [57]. Synthetic CTs can also be created from MRI images acquired at MR-LINAC to allow for MR-only partial breast irradiation [58].

Moreover, online-adaptive radiotherapy has potential in BC as many anatomical changes are observed that are not sufficiently corrected by couch shifts and rotations alone [59]. Galand et al. [60] evaluated the potential benefit of CBCT-based online-adaptation for IMRT of the postoperative breast and lymph node levels. The implemented DL-based autosegmentation had excellent accuracy and the whole adaptive workflow took 5–6 min of additional calculation time not accounting for any required manual editing steps. The feasibility of online-adaptive MR-guided partial breast radiotherapy has already been reported in an early trial of 11 patients with an average in-room treatment time of 26 min [61]. Multiple recruiting prospective trials are currently investigating online-adaptive breast radiotherapy with CBCT and MR-LINAC systems (NCT05727553 and NCT04172753) and the results are pending.

## 5 Artificial intelligence and systemic treatment

### 5.1 Assistance with treatment decisions and guideline-concordant management

In a time of rapidly increasing information, clinicians are looking to AI as support in the decision making for systemic treatment of patients with BC. Bouaud et al. [62] developed a platform that offers several complementary therapeutic decision support modules to improve the quality of care for patients with BC. The platform operates consistently with a common BC knowledge model (BCKM) that follows the generic entity-attribute-value model. The authors introduced the Guideline-Based Decision Support Module (GL-DSS). Three sets of guidelines for BC clinical practice have been structured as decision rules that incorporate levels of evidence, levels of conformance, and two kinds of dependencies (“refinement” and “complement”). When multidisciplinary tumor board (MTB) clinicians changed their decision after using the GL-DSS, it was for a better decision than the decision made without the system in 75 % of the cases [62].

The next option was the Watson for Oncology (WFO) decision system, which has been evaluated for various types of cancer. Liu et al. [63] conducted a study that included patients from northwest China diagnosed with both early-stage (adjuvant setting) and advanced-stage BC. The treatment decisions made by WFO were compared with those made by clinicians. The study found that WFO treatment decisions agreed with the clinicians' decisions 80.2 % of the time for early-stage BC and 50.5 % for advanced-stage BC [63]. Another study by Somashekhar et al. [64] evaluated the concordance between treatment recommendations made by WFO and a MTB for BC. The study was conducted in India and included 638 patients with BC. The results showed that in 93 % of cases, the treatment recommendations made by WFO and the MTB were in agreement. However, recommendations for patients with stage I or IV disease were less likely to be concordant than those for patients with stage II or III disease. Concordance also decreased with increasing age of the patient. On the other hand, Xu et al. [65] examined the impact of a clinical decision support system (CDSS) using WFO on BC treatment decisions and adherence to the National Comprehensive Cancer Center (NCCN) guidelines. The observational study showed that treatment decisions changed in 105 (5 %) of 1977 patients. These changes were primarily seen in patients with HR + disease or those in the first-line therapy setting for stage IV disease, accounting for 73 % and 58 %, respectively. The likelihood of changes in decisions was higher in patients with HR + BC and lower in those diagnosed with stage IIA or IIIA BC. After using the CDSS, there was a slight increase in adherence to the NCCN treatment guidelines [65].

LLMs became widely popular in recent years. Lukac et al. [1] reported a pilot study conducted on 10 consecutive cases of patients with early BC discussed in MTB. The study aimed to evaluate the potential of ChatGPT, in supporting the MTB in planning the therapy of patients with BC. The study found that ChatGPT primarily gave broad responses about chemotherapy, breast surgery, radiotherapy, and antibody therapy. It could recognize risk factors for inherited BC and highlight the older patient who was a candidate for chemotherapy to assess the cost-effectiveness. However, ChatGPT incorrectly classified patients with HER2 1+ and 2+ (FISH negative) as requiring antibody therapy and achieved only 16 % congruence score with the decision of MTB. Similarly, an observational study was conducted by Griewing et al. [66] that aimed to compare the concordance of treatment recommendations from ChatGPT 3.5 with those of an MTB for BC. The results showed that ChatGPT and the MTB agreed on treatment recommendations for half of the patient profiles, including those with precancerous conditions. When evaluating profiles of invasive BC, the agreement rate was 58.8 %. However, the study also noted instances where ChatGPT made significant erroneous decisions. Therefore, the authors concluded that the current development status of publicly available ChatGPT is not adequate as a support tool for MTB [1,66]. Indeed, ChatGPT requires offline training, and because these models have cutoff training dates, they can become outdated with current guidelines by the time they become publicly available.

### 5.2 Predicting response to therapy and outcomes

Achieving pCR is a positive prognostic factor for patients with BC. In triple-negative breast cancer (TNBC), Krishnamurthy et al. [67] conducted a study to develop and validate a deep convolutional NN–based AI model to predict the response to NAC. The researchers used WSIs of hematoxylin and eosin-stained core biopsies to train and validate the model. The study found that predicting response to NAC was possible with the use of AI on digitized images. The AI score's predictive capacity for the whole group showed AUC of 0.751. For stages I, II, and III of the disease, the AUCs were 0.88, 0.73, and 0.74, respectively. With a threshold of 0.35, the AI score had a positive predictive value of 73.7 % for predicting pCR, and a negative predictive value of 76.2 % for predicting non-complete response. In a study evaluating HER2+ and TNBC, Huang et al. [68] developed a WSI feature extraction pipeline called IMage-based Pathological REgistration and Segmentation Statistics (IMPRESS). The MLM developed in this study utilized IMPRESS and clinical features to accurately predict the response to NAC in patients with BC. The results showed that this approach surpassed the outcomes derived from features manually created by pathologists for both HER2+ BC and TNBC.

Another study from Brazil reviewed medical records of 130 patients treated with NAC and including all molecular subtypes of BC [69]. The results showed that the AI network, using only clinicopathologic data, was successful in accurately predicting pCR in 83.3 % of the cases. Additionally, it predicted locoregional recurrence in 95.6 % of the cases and was able to accurately determine the survival status of patients at a specific time point with 90 % accuracy. Moreover, Sammut et al. [70] recognized the need to integrate not only clinical parameters, but also pre-therapy features. The authors demonstrated that combining tumor mutational and copy number landscapes, tumor proliferation, immune infiltration, and T cell dysfunction and exclusion into a multi-omic MLM enabled the model to predict a pCR in patients with BC with an AUC of 0.87.

While these studies show significant promise for predicting response to therapy and outcomes, it is important to consider that these are small studies and ideally the approach should be tested prospectively. In fact, a recent metanalysis raised concerns of bias with the approach of using AI for prediction of treatment outcomes [71]. This stresses the importance that all stakeholders should have deep knowledge of their datasets and their intrinsic biases.

## 6 Supportive therapy

The data about AI in supportive therapy are limited and the present studies focused mostly on chatbots. A randomized controlled trial involving 150 women with BC during chemotherapy in Egypt randomly assigned participants to three groups: ChemoFreeBot, nurse-led education, and routine care [72]. Results have demonstrated that the ChemoFreeBot significantly improved self-care behaviors and reduced the severity, frequency, and distress of chemotherapy side effects compared to the other two groups.

Another study evaluated ChatGPT's responses to both basic and complex oncological questions compared to Google's feature snippet [73]. ChatGPT was able to generate interpretable responses, potentially reducing alarm compared to Google's snippet. Similar results were reported in a study by Lukac et al. comparing answers of ChatGPT, Google and an app about side effects of systemic therapy of BC [74]. ChatGPT's lack of references and potential for incorrect answers leading to harm of the patient should be noted [73]. In this regard, the PERSIST project aims at improving health outcomes, treatment effectiveness, and quality of life of breast and colorectal cancer survivors who have completed their initial treatment through advanced technologies such as Big Data and AI integrated in a mobile health system (mHealthApp), but results are pending [75]. The data about AI in the supportive treatment are limited and AI is not ready yet for the clinical use in this setting.

## 7 Critical aspect to consider before clinical implementation

AI is reshaping cancer care by refining diagnostic accuracy, enhancing prognostic assessments, and personalizing treatment strategies, yet its integration into clinical practice demands an intricate and nuanced approach to address biases that arise throughout the AI lifecycle.

AI tools in clinical practice can be categorized into regulated and non-regulated entities. Regulated AI tools undergo formal review processes by regulatory agencies, such as the U.S. Food and Drug Administration (FDA) or the European Medicines Agency. This process involves extensive documentation, clinical trials and transparency about the dataset used for training and validation. Once approved, the regulated tools are generally considered reliable for clinical use within the scope of their approved applications. On the other hand, non-regulated tools do not undergo formal review by such regulatory entities. Therefore, while often innovative, their clinical use carries risks such as potential inaccuracies, unvalidated performance claims, and unchecked biases that could have a negative impact on patient outcomes.

It is important to stress that AI algorithms are not FDA-approved in the same way drugs are. Instead, most algorithms are merely FDA cleared under Section 510(k). Such considerations are crucial due to the potential lack of access to the patient population data on which the algorithm was trained and tested. Moreover, AI built on a poor dataset, with limited granularity and biased feature distributions carries the intrinsic biases of the dataset [76].

In oncology, the initial formulation of the task and the subsequent selection of data are critical junctures where bias can infiltrate the system. Historical bias, for example, is embedded in legacy datasets that reflect longstanding disparities in healthcare access and outcomes. These data often mirror systemic inequalities that, if uncorrected, cause AI models to replicate rather than rectify past prejudices. Equally important is representation bias, which occurs when the data used for training do not accurately capture the diversity of the intended patient population. This bias leads to models that may perform adequately in well-represented groups yet fail to generalize to underrepresented populations, thereby exacerbating inequities in care delivery [77].

Moreover, sampling bias further complicates model reliability when data collection methods inadvertently favor certain subgroups over others, resulting in non-random samples that skew the learning process. Omitted variable bias presents another challenge when critical clinical factors—such as prior treatment history, comorbidities, or nuanced biomarker profiles—are excluded from the model input. Excluding these factors impairs the predictive power and clinical relevance of the AI tool. Measurement bias can also distort outcomes. Variations in data acquisition protocols, imaging standards, or even differences in how clinical endpoints are defined and recorded can lead to inconsistencies that compromise model accuracy. Similarly, labeling bias emerges from the subjective nature of data annotation. Human factors and varying interpretative standards in assigning clinical labels can introduce errors that propagate through the algorithm [76]. These biases are summarized in Table 1.

**Table 1:** Summary of main biases.

Type of bias	Description
Historical bias	Presence of systemic inequalities embedded in legacy data
Representation bias	Inadequate diversity representation in training data
Sampling bias	Non-random sample collection favoring certain groups
Omitted variable bias	Exclusion of clinically significant predictive factors
Measurement bias	Inconsistencies in data acquisition and clinical endpoints
Labeling bias	Errors from subjective annotation and varied standards

Addressing these biases requires a comprehensive strategy that spans data curation, model development, and post-deployment validation [78]. Advanced data preprocessing techniques—such as multiple imputation for handling missing values, robust outlier detection, and corrective measures to rebalance skewed datasets—are essential to ensure that the training data faithfully represent the heterogeneity of real-world clinical scenarios. Debiasing methodologies, including reweighting and resampling strategies, as well as fairness-aware regularization during model training are vital to counteract the influence of spurious correlations and indirect discrimination. In addition, the use of interpretable models is paramount. By designing algorithms that not only perform well but also provide transparent rationales for their predictions, clinicians can more readily scrutinize and validate the AI outputs. This mitigates risks associated with confirmation and automation biases that may lead to overreliance on algorithmic recommendations [71].

The integration of interdisciplinary teams is another cornerstone in this process. Combining the expertise of oncologists, data scientists, statisticians, and social scientists fosters a multifaceted understanding of both the clinical and technical dimensions of bias. Such collaborative efforts ensure that feature selection is guided by clinical relevance and that the algorithms are continuously refined through rigorous local validation and recalibration. Given that AI performance may drift over time—owing to shifts in patient demographics, evolving clinical practices, or changes in imaging technology—continuous monitoring becomes indispensable. This iterative process of model auditing and real-time adjustment not only safeguards against degradation in performance but also adapts to the dynamic nature of clinical environments.

Furthermore, operational strategies such as federated learning offer promising avenues to tailor AI systems to the unique characteristics of local populations while preserving data privacy. By enabling decentralized training on diverse datasets, federated learning can mitigate geographical and infrastructural biases, ensuring that AI tools remain robust across different healthcare settings. Finally, the AI performance can vary significantly between hospitals once deployed and can drop over time due to dataset shift, calibration drift, and similar phenomena [76,79]. This requires a skilled hybrid team of clinicians, data scientists, and engineers to periodically monitor and verify the quality of the algorithm's performance.

The convergence of these technical and organizational strategies paves the way for AI to fulfill its promise in oncology—not only by enhancing the precision of diagnostics and the personalization of therapy but also by advancing the broader goal of equitable, high-quality cancer care.

## 8 Conclusion

AI is progressively advancing BC care. Despite positive achievements in recent years, as a treatment support it is currently only used in RT for organ-at-risk contouring, target volume contouring and online-adaptive RT treatment. The future potential could be expected in the field of radiation oncology and might involve automated RT treatment planning, image-guided RT delivery and outcome prediction after neoadjuvant treatment. Furthermore, surgery will likely benefit from early AI implementation for breast reconstruction planning, intraoperative margins evaluation, and prediction of postoperative complications. There are some AI applications that currently remain far from clinical usability. These include AI-assisted treatment decision making regarding systemic treatment, support to omit breast or axillary surgery after neoadjuvant treatment, and patient support during and after treatment. In the future, the prospective validation and post-deployment monitoring are crucial to maintain high-quality standards in breast cancer care accompanied with AI, and ultimately may lead to an increase in the quality of personalized health care and resource optimization.

## CRediT authorship contribution statement

**Stefan Lukac:** Writing – review & editing, Writing – original draft, Validation, Methodology, Investigation, Formal analysis, Data curation, Conceptualization. **Florian Putz:** Writing – review & editing, Writing – original draft, Validation, Investigation, Formal analysis. **Giacomo De Micheli:** Writing – review & editing, Writing – original draft, Validation, Methodology, Investigation, Formal analysis, Conceptualization. **Chiara Corti:** Writing – review & editing, Writing – original draft, Validation, Investigation, Formal analysis. **Wolfgang Janni:** Writing – review & editing, Supervision, Investigation. **Sara M. Tolaney:** Writing – review & editing, Supervision, Investigation. **Giuseppe Curigliano:** Writing – review & editing, Supervision, Investigation. **Sibylle Loibl:** Writing – review & editing, Supervision, Investigation. **Paolo Tarantino:** Writing – review & editing, Validation, Methodology, Investigation, Conceptualization. **Jose Pablo Leone:** Writing – review & editing, Writing – original draft, Validation, Methodology, Investigation, Formal analysis, Data curation, Conceptualization.

## Disclosures

**PT** reports research funds (to institution) from AstraZeneca, consulting/advisory role for AstraZeneca, Daiichi Sankyo, Novartis, Gilead, Roche, Genentech, Eli Lilly, BioThera. **CCo** reports travel/accommodations (to scientific meeting) from Veracyte and is supported by the Fondazione Gianni Bonadonna (FGB) and Associazione Italiana per la Ricerca contro il Cancro (AIRC) (2024–2026). **SMT** reports research funds (to institution) from Genentech/Roche, Merck, Exelixis, Pfizer, Lilly, Novartis, Bristol Myers Squibb, Eisai, AstraZeneca, Gilead, NanoString Technologies, SeaGen, OncoPep, Daiichi Sankyo, Menarini/Stemline, Jazz Pharmaceuticals; consulting/advisory role for Novartis, Pfizer/SeaGen, Merck, Eli Lilly, AstraZeneca, Genentech/Roche, Eisai, Bristol Myers Squibb/Systimmune, Daiichi Sankyo, Gilead, Blueprint Medicines, Reveal Genomics, Sumitovant Biopharma, Artios Pharma, Menarini/Stemline, Aadi Bio, Bayer, Jazz Pharmaceuticals, Natera, Tango Therapeutics, eFFECTOR, Hengrui USA, Cullinan Oncology, Circle Pharma, Arvinas, BioNTech, Johnson&Johnson/Ambrx, Launch Therapeutics, Zuellig Pharma, Bicycle Therapeutics, BeiGene Therapeutics, Mersana, Summitt Therapeutics, Avenzo Therapeutics, Aktis Oncology, Celcuity, Boehringer Ingelheim, Samsung Bioepis, Olema Pharmaceuticals; and travel support from Eli Lilly, Gilead, Jazz Pharmaceuticals, Pfizer, Arvinas, Roche. All the competing interests were outside the submitted work.

## Funding

None.

## Declaration of competing interest

The authors declare the following financial interests/personal relationships which may be considered as potential competing interests: Paolo Tarantino reports a relationship with AstraZeneca that includes: consulting or advisory and funding grants. Paolo Tarantino reports a relationship with Daiichi Sankyo that includes: consulting or advisory. Paolo Tarantino reports a relationship with Novartis that includes: consulting or advisory. Paolo Tarantino reports a relationship with Gilead that includes: consulting or advisory. Paolo Tarantino reports a relationship with Roche that includes: consulting or advisory. Paolo Tarantino reports a relationship with Genentech that includes: consulting or advisory. Paolo Tarantino reports a relationship with Eli Lilly that includes: consulting or advisory. Paolo Tarantino reports a relationship with Biothera that includes: consulting or advisory. Chiara Corti reports a relationship with Veracyte Inc that includes: travel reimbursement. Chiara Corti reports a relationship with Fondazione Gianni Bonadonna that includes: funding grants. Chiara Corti reports a relationship with Associazione Italiana per la Ricerca contro il Cancro that includes: funding grants. Sara M. Tolaney reports a relationship with Genentech that includes: consulting or advisory and funding grants. Sara M. Tolaney reports a relationship with Merck & Co Inc that includes: consulting or advisory and funding grants. Sara M. Tolaney reports a relationship with Exelixis Inc that includes: funding grants. Sara M. Tolaney reports a relationship with Pfizer that includes: consulting or advisory and funding grants. Sara M. Tolaney reports a relationship with Eli Lilly and Company that includes: consulting or advisory, funding grants, and travel reimbursement. Sara M. Tolaney reports a relationship with Novartis that includes: consulting or advisory and funding grants. Sara M. Tolaney reports a relationship with Bristol-Myers Squibb Company that includes: consulting or advisory and funding grants. Sara M. Tolaney reports a relationship with AstraZeneca that includes: consulting or advisory and funding grants. Sara M. Tolaney reports a relationship with NanoString Technologies Inc that includes: funding grants. Sara M. Tolaney reports a relationship with Gilead Sciences Inc that includes: consulting or advisory, funding grants, and travel reimbursement. Sara M. Tolaney reports a relationship with Seagen Inc that includes: consulting or advisory and funding grants. Sara M. Tolaney reports a relationship with OncoPep that includes: funding grants. Sara M. Tolaney reports a relationship with Daiichi Sankyo Inc that includes: consulting or advisory and funding grants. Sara M. Tolaney reports a relationship with Menarini Stemline UK Limited that includes: consulting or advisory and funding grants. Sara M. Tolaney reports a relationship with Jazz Pharmaceuticals Inc that includes: consulting or advisory, funding grants, and travel reimbursement. Sara M. Tolaney reports a relationship with Eisai Inc that includes: consulting or advisory. Sara M. Tolaney reports a relationship with Blueprint Medicines Corporation that includes: consulting or advisory. Sara M. Tolaney reports a relationship with Reveal Genomics SL that includes: consulting or advisory. Sara M. Tolaney reports a relationship with Sumitovant BioPharma Inc that includes: consulting or advisory. Sara M. Tolaney reports a relationship with Artios Pharma Ltd that includes: consulting or advisory. Sara M. Tolaney reports a relationship with Aadi Bio that includes: consulting or advisory. Sara M. Tolaney reports a relationship with Bayer Corporation that includes: consulting or advisory. Sara M. Tolaney reports a relationship with Natera, Inc. that includes: consulting or advisory. Sara M. Tolaney reports a relationship with Tango Therapeutics Inc that includes: consulting or advisory. Sara M. Tolaney reports a relationship with eFFECTOR Therapeutics Inc that includes: consulting or advisory. Sara M. Tolaney reports a relationship with Hengrui USA that includes: consulting or advisory. Sara M. Tolaney reports a relationship with Cullinan Oncology that includes: consulting or advisory. Sara M. Tolaney reports a relationship with Circle Pharma that includes: consulting or advisory. Sara M. Tolaney reports a relationship with Arvinas Inc that includes: consulting or advisory and travel reimbursement. Sara M. Tolaney reports a relationship with BioNTech US Inc that includes: consulting or advisory. Sara M. Tolaney reports a relationship with Launch Therapeutics that includes:. Sara M. Tolaney reports a relationship with Zuellig Pharma Holdings Pte Ltd that includes: consulting or advisory. Sara M. Tolaney reports a relationship with Johnson & Johnson that includes: consulting or advisory. Sara M. Tolaney reports a relationship with Bicycle Therapeutics plc that includes: consulting or advisory. Sara M. Tolaney reports a relationship with BeiGene that includes: consulting or advisory. Sara M. Tolaney reports a relationship with Mersana Therapeutics Inc that includes: consulting or advisory. Sara M. Tolaney reports a relationship with Summit Therapeutics plc that includes: consulting or advisory. Sara M. Tolaney reports a relationship with Avenzo Therapeutics that includes: consulting or advisory. Sara M. Tolaney reports a relationship with Aktis Oncology that includes: consulting or advisory. Sara M. Tolaney reports a relationship with Celcuity, Inc. that includes: consulting or advisory. Sara M. Tolaney reports a relationship with Boehringer Ingelheim Corp USA that includes: consulting or advisory. Sara M. Tolaney reports a relationship with Samsung Bioepis.Co., Ltd. that includes: consulting or advisory. Sara M. Tolaney reports a relationship with Olema Pharmaceuticals, Inc. that includes: consulting or advisory and funding grants. Sara M. Tolaney reports a relationship with Roche that includes: travel reimbursement. If there are other authors, they declare that they have no known competing financial interests or personal relationships that could have appeared to influence the work reported in this paper.
